# Large Ovarian Cystadenoma in an Adolescent Girl: A Case Report

**DOI:** 10.7759/cureus.43108

**Published:** 2023-08-08

**Authors:** Fahad N Alsolami, Layan S Alfraidi, Ibrahim M Alharbi, Sherefah I Alsayafi, Ahlam Alharbi

**Affiliations:** 1 General Practice, Dar Al Uloom University, Riyadh, SAU; 2 Family Medicine, Primary Health Care Center, Riyadh, SAU

**Keywords:** case report, computed tomography, adolescence, acute abdominal pain, ovarian cystadenoma

## Abstract

Ovarian cystadenomas are rare neoplastic tumors arising from the ovarian surface epithelium. While commonly observed in adult women, their occurrence in adolescents is exceedingly uncommon. The management of large ovarian cystadenomas in this age group poses unique challenges due to acute presentations and potential complications. We present the case of a 16-year-old girl who presented with sudden, severe abdominal pain and distension. Imaging revealed a 15 cm complex cystic mass originating from the right ovary, consistent with a cystadenoma. Urgent surgical intervention led to the right salpingo-oophorectomy, confirming the benign nature of the tumor. This report highlights the importance of a comprehensive approach to diagnosing and managing rare ovarian neoplasms in adolescents. Timely recognition, appropriate imaging, histopathological evaluation, and surgical intervention are crucial for optimal outcomes and reducing potential complications.

## Introduction

Ovarian cystadenomas are neoplastic tumors that arise from the surface epithelium of the ovaries. They represent a common subset of ovarian neoplasms and can be classified into serous, mucinous, endometrioid, or clear cell subtypes [1]. Ovarian cystadenomas are predominantly found in adult women, and their occurrence in the adolescent population is exceedingly rare. Although specific epidemiological data on the incidence or prevalence of large ovarian cystadenomas in adolescents is limited, published literature suggests that these tumors are uncommon in this age group. The rarity of large ovarian cystadenomas in adolescents can present unique challenges in diagnosis and management, necessitating a comprehensive approach to care. These tumors often manifest with acute and severe symptoms, necessitating urgent medical attention and surgical intervention [1,2]. Prompt diagnosis and appropriate management are crucial to prevent complications such as ovarian torsion, rupture, or peritonitis [3]. In this report, we present a case of an adolescent girl with a large ovarian cystadenoma, which led to an emergency presentation due to the sudden onset of severe abdominal pain and distension.

## Case presentation

We present the case of a 16-year-old adolescent girl who presented to the emergency department with severe abdominal pain and distension. The patient, previously healthy, had no significant medical history or known risk factors. She was not on any medications and had no history of surgical interventions. The patient's menstrual cycles had been regular, and there were no reported gynecological abnormalities or symptoms prior to the current presentation.

The patient's symptoms began insidiously one week before presentation, characterized initially by mild lower abdominal discomfort, which gradually progressed to severe, sharp pain. In the preceding 24 hours, she noticed a rapid increase in abdominal girth, leading to significant distension. The pain became excruciating, prompting her parents to seek immediate medical attention.

On examination, the patient appeared in acute distress, with a pallor complexion. Her vital signs were within normal limits, except for a slightly elevated heart rate of 100 beats per minute. Abdominal examination revealed a tense, distended abdomen with diffuse tenderness and guarding. A large, firm, and mobile mass was palpated in the lower abdomen, extending above the umbilicus. Bowel sounds were present but diminished.

The initial work-up included routine blood tests, which revealed a mild leukocytosis (white blood cell count: 12,500/µL, normal range: 4,500-11,000/µL) and an elevated C-reactive protein level of 25 mg/L (normal range: 0-5 mg/L). Serum levels of tumor markers, including alpha-fetoprotein and beta-human chorionic gonadotropin, were within normal limits. To further evaluate the large ovarian mass and assess its characteristics, a contrast-enhanced computed tomography scan of the abdomen and pelvis was performed urgently.

**Figure 1:**
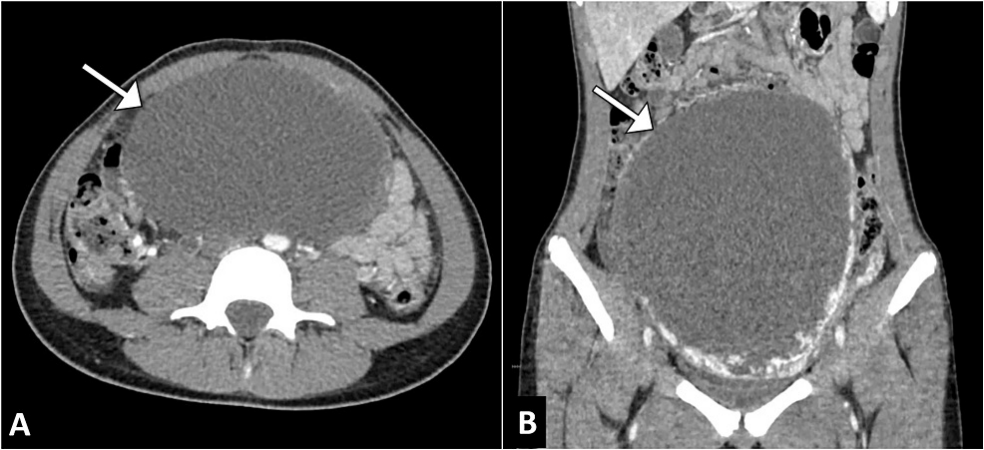
Axial (A) and coronal (B) CT images of the abdomen demonstrating a large unilocular abdominopelvic cystic mass (arrow) causing mass effect by displacing adjacent bowel loops.

The computed tomography scan revealed a large, well-defined cystic mass originating from the right ovary. The mass measured approximately 15 cm in its greatest dimension and displayed peripheral areas of calcification along with multiple internal septations, enhancing solid components. The findings were consistent with a complex ovarian cystadenoma of the right ovary (Figure 1).

Given the patient's acute presentation, the size of the ovarian mass, and the potential for complications such as ovarian torsion or rupture, urgent surgical intervention was deemed necessary. The patient was counseled, and informed consent was obtained from her parents. The gynecological surgical team performed an exploratory laparotomy.

**Figure 2:**
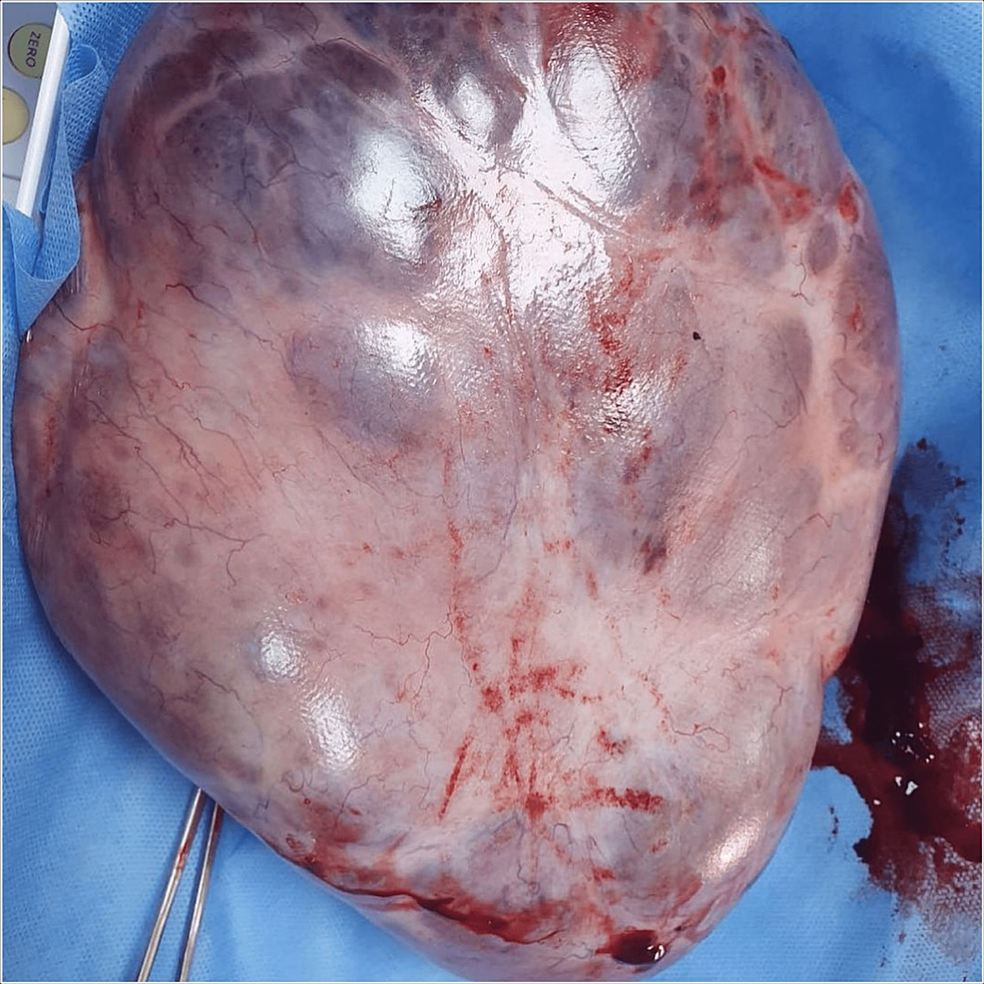
Gross pathology image of the resected large ovarian cystic mass.

Intraoperatively, a large right ovarian cystadenoma was identified, along with evidence of ovarian torsion (Figure 2). Due to compromised blood flow and the risk of further complications, a right salpingo-oophorectomy was performed. The ovarian mass was carefully excised, and intraoperative frozen section analysis confirmed the benign nature of the tumor.

**Figure 3:**
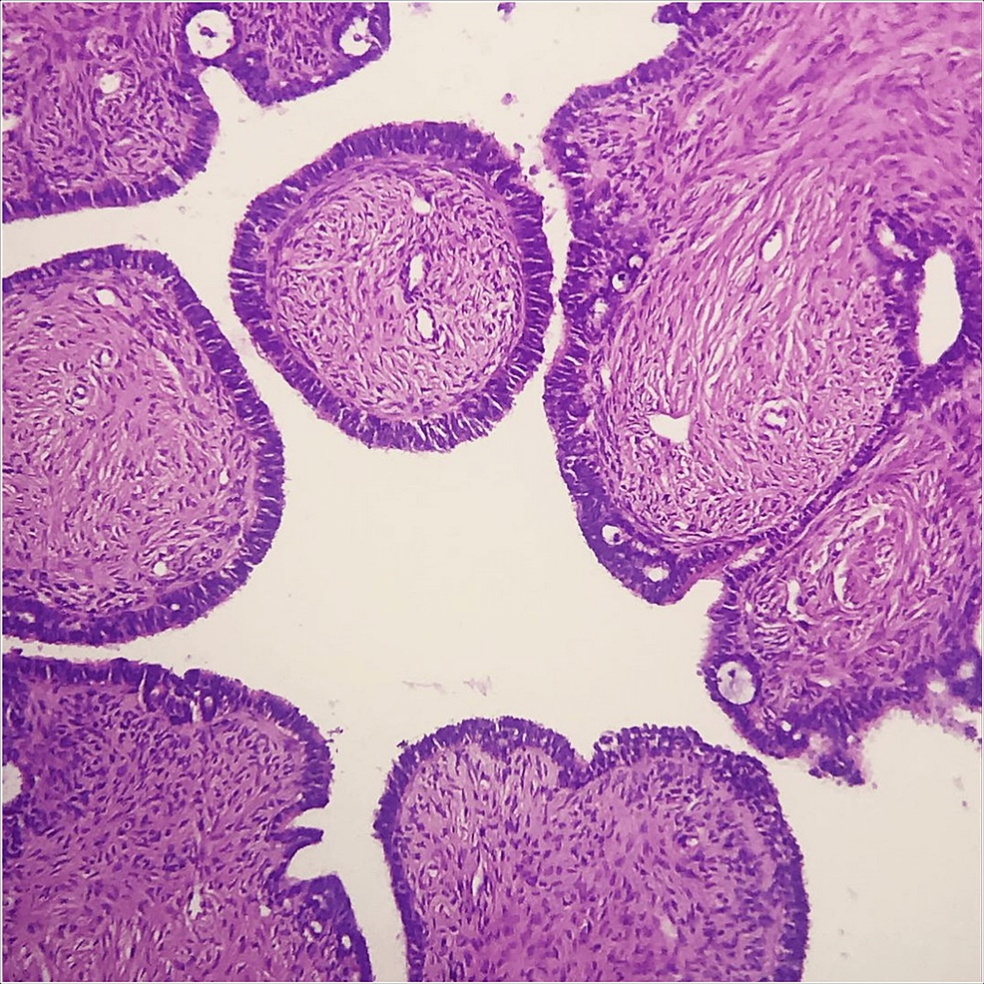
Histopathological image showing unilocular cysts lined by ciliated pseudostratified cuboidal epithelium, consistent with ovarian cystadenoma.

Histopathology examination of the excised mass revealed a unilocular cystic structure lined by columnar epithelium with focal areas of papillary projections. The stroma showed mild cellular proliferation and areas of calcification, consistent with the diagnosis of a benign ovarian cystadenoma (Figure 3).

Postoperatively, the patient's recovery was uneventful. She received appropriate pain management and was closely monitored in the recovery unit. The patient's condition steadily improved, and she was able to tolerate oral intake without any complications. The patient was discharged on the fourth postoperative day in stable condition. She was provided with detailed instructions for wound care and advised to follow up with the gynecological team for regular check-ups.

## Discussion

The patient in this case exhibited an acute presentation with severe abdominal pain and distension, prompting immediate medical attention. The rapid increase in abdominal girth and the presence of a palpable mass raised concerns about a potential ovarian neoplasm [4]. Differential diagnosis of ovarian masses in adolescents includes benign lesions such as ovarian cysts, cystadenomas, and teratomas, as well as malignant tumors like ovarian germ cell tumors and sex cord-stromal tumors. Other conditions, such as ectopic pregnancy and tubo-ovarian abscess, were also considered. The urgency of the situation necessitated a comprehensive diagnostic work-up to identify the underlying pathology accurately [1].

Imaging studies, particularly the contrast-enhanced computed tomography scan, played a crucial role in characterizing the ovarian mass in this case. The computed tomography scan revealed a large, complex cystic mass originating from the right ovary, with internal septations, solid components, and calcification, consistent with a cystadenoma. This information guided the surgical approach and informed the decision for a right salpingo-oophorectomy, which was confirmed by intraoperative frozen section analysis. Histopathology further supported the benign nature of the tumor, guiding the subsequent management and providing crucial information for prognostication [5].

While large ovarian cystadenomas are relatively rare in adolescents, there have been sporadic case reports in the medical literature [4,5]. The age of presentation and the sudden onset of symptoms, in this case, align with previous reports, highlighting the potential for acute and dramatic clinical presentations of ovarian cystadenomas in this age group [1]. Additionally, the size and complexity of the cystadenoma, in this case, are consistent with the findings in the literature [5-7], further validating the significance of early diagnosis and prompt surgical intervention [3].

The emergent nature of the presentation and the potential for complications necessitated urgent surgical intervention in this case. The decision for a right salpingo-oophorectomy was made due to the evidence of ovarian torsion, compromising blood flow to the affected ovary [3]. The decision for a right salpingo-oophorectomy was made due to the evidence of ovarian torsion, compromising blood flow to the affected ovary, necessitating urgent removal to prevent further complications. If there had been no ovarian torsion, alternative surgical approaches, such as ovarian cystectomy or cystadenoma enucleation, might have been considered, taking into account the specific characteristics of the cystadenoma and the patient's individual health and fertility considerations. The surgical approach aimed to achieve complete excision of the tumor while preserving the viability of the surrounding tissues. A multidisciplinary approach, involving gynecologists, surgeons, and pathologists, was crucial in achieving a favorable outcome for the patient [1].

## Conclusions

The presented case of a large ovarian cystadenoma in an adolescent girl underscores the importance of a systematic and comprehensive approach to the diagnosis and management of rare ovarian neoplasms in this age group. Timely recognition of acute presentations, appropriate imaging studies, histopathological evaluation, and surgical intervention are vital in achieving successful outcomes and minimizing potential complications.
